# Peer Review of “Effects of Pharmacogenomic Testing in Clinical Pain Management: Retrospective Study”

**DOI:** 10.2196/37513

**Published:** 2022-05-03

**Authors:** Madara Hetti Arachchilage

**Affiliations:** 1 Zoetis Inc Urbana, IL United States

**Keywords:** pharmacogenomics, pain management, drug-drug interaction, DDI, pharmacy, prescriptions, genetics, genomics, drug-gene interaction, pain

*This is a peer-review report submitted for the paper “Effects of Pharmacogenomic Testing in Clinical Pain Management: Retrospective Study.”*

## Review Round 1

### General Comments

Authors of this manuscript [1] have determined the impact of pharmacogenetic (PGx) testing on pain medication prescribing. A retrospective analysis was conducted with 171 patients in a pain management clinic during 2016 to 2018 within the western United States. A novel deep sequencing (>1000X) PGx panel is described encompassing 23 genes combined with PGx dosing guidance, drug-gene interaction, and drug-drug interaction reporting to prevent adverse drug reaction events. This manuscript is interesting and well-written. However, the Methods and Discussion section of the manuscript could be improved for clarity. Please refer to my comments below.

### Specific Comments

#### Major Comments

##### Abstract

1. What was the primary outcome of this study? Is it to report the number of cases where PGx information could be used to optimize drug dosing?

2. “This study demonstrates a successful implementation of PGx testing utilizing an extended PGx panel combined with a customized, informational report to help improve clinical outcomes.” Did authors develop a software platform to generate a customized, informational report to help improve clinical outcomes? I do not see any discussion on this matter. What were the parameters of the effectiveness and safety of treatment in evaluated patients? Did you do any statistical testing to find an association between the presence of a polymorphic gene variant and the impact of pharmacotherapy? Did you have a control group?

##### Introduction

3. I would be interested in having a brief introduction to currently available PGx panels, and what the strengths of the panel in this study are.

##### Methods

4. “23 genes were selected based on having the most clinical utility in PGx at the time of design in April 2016 (ADRA2A, CES1, COMT, CYP1A2, CYP2C19, CYP2C9, CYP2D6, CYP3A4, CYP3A5, DRD1, DRD2, F2, F5, GNB3, HTR1A, HTR2A, HTR2C, MTHFR, OPRM1, SLC6A2, SCL6A4, SLCO1B1, VKORC1).” What were the criteria used to narrow down genes that authors considered of most clinical utility in PGx?

5. “75 target regions were covered by 82 amplicons with an average amplicon size of 250 base pairs (bp)” Can you elaborate on 75 target regions? Did the authors have multiple target regions per gene? If so, details should be provided.

6. What were the medical conditions of patients with pain management in this study? Was it varied across patients in this cohort? I would like to see the authors’ discussion on this.

7. “PGx reporting were obtained retrospectively from patients (n = 171) in a pain management clinic representing an ethnically diverse patient population from 2016 to 2018 within the western United States.” Although authors report that they have an ethnically diverse patient population, no descriptive statistics on demographics, age, and clinical information was provided.

8. What factors were tested on urine toxicology and progress report?

##### Results and Discussion

9. While the manuscript describes 3 patients (patient A, B, and C) who did not stick to the treatment regimen and drug response adversaries, did patients who stuck to treatment regimens based on PGx testing show any side effects or did they do any survey for reporting pain symptoms? For example, were they tested for adverse drug reactions or partial or complete response to treatments?

10. I would like to see a discussion on key limitations of this study and further improvement on this study.

11. Have you looked into genotype frequencies of different ethnic populations in your study? What benefits do you anticipate by studying PGx-guided treatment interventions on diverse ethnic populations?

##### Conclusion

12. “This study demonstrates the predictive value of PGx testing combined with a customized informational report to help improve clinical outcomes, which resulted in increased utilization on patients in a pain management setting.” On what basis do the authors claim increased utilization on patients in a pain management setting? Did you do any statistical analysis to back up this statement?

## Abbreviations

PGx: pharmacogenetic
